# Acute kidney injury adversely affects the clinical course of acute myeloid leukemia patients undergoing induction chemotherapy

**DOI:** 10.1007/s00277-021-04482-3

**Published:** 2021-03-11

**Authors:** Olivier Ballo, Fagr Eladly, Stefan Büttner, Jan Alexander Stratmann, Sarah Rudolf, Uta Brunnberg, Eva-Maria Kreisel, Björn Steffen, Sebastian Wagner, Fabian Finkelmeier, Hubert Serve, Christian H. Brandts

**Affiliations:** 1 Department of Medicine, Hematology/Oncology, University Hospital, Goethe University, Frankfurt, Germany; 2 Department of Medicine, Nephrology, University Hospital, Goethe University, Frankfurt, Germany; 3 German Cancer Consortium (DKTK) and German Cancer Research Center (DKFZ), Heidelberg, Germany; 4 Department of Medicine, Gastroenterology, Hepatology and Endocrinology, University Hospital, Goethe University, Frankfurt, Germany; 5 University Cancer Center Frankfurt (UCT), University Hospital, Goethe University, Frankfurt, Germany

**Keywords:** Acute kidney failure, Acute myeloid leukemia, Induction chemotherapy, Survival, Intensive care treatment

## Abstract

Acute kidney injury (AKI) complicates the clinical course of hospitalized patients by increasing need for intensive care treatment and mortality. There is only little data about its impact on AML patients undergoing intensive induction chemotherapy. In this study, we analyzed the incidence as well as risk factors for AKI development and its impact on the clinical course of AML patients undergoing induction chemotherapy. We retrospectively analyzed data from 401 AML patients undergoing induction chemotherapy between 2007 and 2019. AKI was defined and stratified according to KIDGO criteria by referring to a defined baseline serum creatinine measured on day 1 of induction chemotherapy. Seventy-two of 401 (18%) AML patients suffered from AKI during induction chemotherapy. AML patients with AKI had more days with fever (7 vs. 5, *p* = 0.028) and were more often treated on intensive care unit (45.8% vs. 10.6%, *p* < 0.001). AML patients with AKI had a significantly lower complete remission rate after induction chemotherapy and, with 402 days, a significantly shorter median overall survival (OS) (median OS for AML patients without AKI not reached). In this study, we demonstrate that the KIDGO classification allows mortality risk stratification for AML patients undergoing induction chemotherapy. Relatively mild AKI episodes have impact on the clinical course of these patients and can lead to chronic impairment of kidney function. Therefore, we recommend incorporating risk factors for AKI in decision-making considering nutrition, fluid management, as well as the choice of potentially nephrotoxic medication in order to decrease the incidence of AKI.

**Supplementary Information:**

The online version contains supplementary material available at 10.1007/s00277-021-04482-3.

## Introduction

Acute myeloid leukemia (AML) is a hematological malignancy arising from a clonal proliferation of myeloid precursors losing their ability to differentiate into mature functional blood cells. A curative therapy approach can only be achieved by intensive induction chemotherapy. During the past 30 years, advances in intensified chemotherapy, allogenic stem cell transplantation (SCT), and supportive measures resulted in increased survival rates [1, 2]. Still, AML patients undergoing induction chemotherapy are at high risk to develop complications and treatment-related mortality (TRM) remains at a level of 8–15% [3, 4].

Acute kidney injury (AKI) is defined by a rapid and partly reversible restriction of kidney function causing a reduction of renal glomerular filtration rate (GFR) [5]. AKI has been shown to be associated with increased mortality and a higher rate of treatment on intensive care units (ICU) in hospitalized patients [5, 6]. In pediatric AML patients undergoing intensive chemotherapy, AKI has been correlated to volume depletion, sepsis, and nephrotoxic medications, and a higher risk for developing chronic kidney disease later [7]. Pediatric AML patients with AKI showed a significantly higher mortality rate than AML patients without AKI. Lahoti et al. revealed that 36% of adult AML patients develop AKI during chemotherapy [8]. Advanced age, mechanical ventilation, vasopressors, low WBC, hypoalbuminemia, use of vancomycin, diuretics, and liposomal amphotericin B were positively associated with the occurrence of AKI. Furthermore, the probability of achieving a complete remission (CR) was shown to be significantly lower for AML patients who developed AKI compared to those without AKI. To our knowledge, only the study by Lahoti et al. have investigated the impact of AKI in adult AML patients undergoing intensive chemotherapy.

We conducted this retrospective study to determine the incidence of AKI as defined and classified by *Kidney Disease: Improving Global Outcomes* (KDIGO) *Clinical Practice Guideline* in AML patients undergoing induction chemotherapy at our institute (https://kdigo.org/wp-content/uploads/2016/10/KDIGO-2012-AKI-Guideline-English.pdf). Furthermore, we aimed at identifying potential risk factors for AKI as well as associated clinical conditions.

## Materials and methods

### Study design and treatment protocols

In this single-center study, we retrospectively included all patients aged ≥ 18 with AML (excluding acute promyelocytic leukemia) who underwent intensive induction chemotherapy between 2007 and 2019. AKI was defined and stratified as recommended by the KDIGO *Clinical Practice Guideline* (https://kdigo.org/wp-content/uploads/2016/10/KDIGO-2012-AKI-Guideline-English.pdf). AML patients with an increase in serum creatinine (SCr) by ≥ 0.3 mg/dl within 48 h or an increase in SCr to ≥ 1.5 times baseline within the prior 7 days during the hospital stay of induction chemotherapy were defined as AML patients with AKI. SCr increase 1.5–1.9 times baseline or ≥ 0.3mg/dl was defined as AKI stage 1, and SCr 2.0–2.9 times baseline was defined as AKI stage 2 and SCr 3.0 times baseline or increase in serum creatinine to ≥ 4.0 mg/dl or initiation of renal replacement therapy was defined as AKI stage 3 (Table S1). All other patients were defined as AML patients without AKI. The baseline SCr was defined as the value measured at day 1 of induction chemotherapy. AML patients with a baseline SCr ≥ 1.5 mg/dl were excluded from this study. Urine output was not considered for definition and stratification of AKI. Standard induction chemotherapy was the so-called 7+3-regime; cytarabine 100 mg/m^2^ given intravenous (IV) continuously for 7 days is combined with daunorubicin 60mg/m^2^ given as a 30-min IV infusion on days 3, 4, and 5 [9]. Patients under the age of 60 received a second induction therapy with 7+3 if early blast clearance was achieved in d15 bone marrow blood evaluation or with HAM protocol (cytarabine 3000 mg/m^2^ was administered by 3-h IV infusion every 12 h on day 1 through 3 and mitoxantrone 10 mg/m^2^ by 30-min IV infusion on days 3, 4, and 5) if blast clearance was not achieved on d15 bone marrow blood evaluation [10]. Patients above the age of 60 received only a second induction chemotherapy with HAM (with reduced cytarabine dose of 1000 mg/m^2^), if the first induction therapy cycle was not sufficient to achieve bone marrow blast clearance on d15 [11]. In case of a complete remission (CR) after induction chemotherapy with 7+3 alone or with 7+3 and HAM, patients went on to receive a consolidation treatment with either high-dose cytarabine or with an allogenic SCT. Response assessment was performed in accordance with the European Leukemia Net (ELN) recommendations from 2010 [12]. Patients received routinely antimicrobial prophylaxis with levofloxacin and posaconazole daily as suggested by current guidelines [13, 14]. A day with feve*r* was defined as a body temperature increase above ≥ 38.3 °C once or ≥ 38.0 °C on two consecutive days [15]. If fever or a significant increase of C-reactive protein (CRP) (doubling of CRP level and absolute value above 5 mg/dl, norm < 0.5 mg/dl) was found, antibiotic prophylaxis was replaced by intravenous broad-spectrum antibiotics. Blood testing (hematology, liver and kidney function, coagulation, inflammation markers) was performed every other day routinely.

The study was performed in accordance with the 2013 Helsinki declaration. Patients provided informed written consent to retrospective data extraction from patient charts and patient data was provided after approval by the local Ethics Committee (approval number SHN-01-2020). The ethics committee waived the requirement for informed consent for deceased patients. In addition, the majority of patients were also enrolled in the AML registry of the Study Alliance Leukemia (approval number EK 98032010). After ethics approval, patient data was retrieved from the clinical cancer registry of the University Cancer Center (UCT) Frankfurt, complemented by data directly from the medical records and fully anonymized. Data analysis was performed on anonymized data.

### Statistical analysis

This study was designed as a retrospective cohort study. Patients were followed until death or last contact. Dates of treatment start and finish with induction chemotherapy were assessed separately. Continuous variables are shown as median ± range and categorical variables are reported as frequencies and percentages. All continuous variables were tested for normality and were analyzed by using the Student’s *t*-test or the Wilcoxon-Mann-Whitney test accordingly. Chi-squared test was used for binary variables. Death rates were analyzed by Kaplan-Meier method and curves were compared by log-rank test. Predictors of survival were determined using a univariate Cox regression hazard model. Death was recorded as an event. Statistical analysis was performed with SPSS (Version 22.0, IBM, Armonk, NY, USA).

## Results

Four hundred one patients diagnosed with AML between 2007 and 2019 that underwent intensive induction therapy were included in this retrospective analysis. According to the KDIGO classification, 72 (18%) AML patients suffered from AKI during the hospital stay of induction chemotherapy and were assigned to the AML cohort with AKI, and 329 (82%) AML patients did not suffer from AKI during induction chemotherapy and were assigned to the AML cohort without AKI. SCr increase defining AKI was evaluated with respect to a baseline SCr measured at day 1 of induction chemotherapy. Twenty-two AML patients with a baseline SCr ≥ 1.5 were excluded from further analysis. Urine output was not considered for definition and stratification of AKI.

### Baseline characteristics of AML patients with and without AKI

Median age was 61 years (range 24–78) in AML patients with AKI and 58 years (range 18–82) in AML patients without AKI (*p* = 0.014) (Table 1). The fraction of AML patients older than 60 years of age was 51.4% in AML patients with AKI and 41.6% in AML patients without AKI (*p* = 0.149). The median albumin level on the day of admission was 3.7 g/dl (2–4.9) in AML patients with AKI and 3.8 g/dl (2–5.2) in AML patients without AKI (*p* = 0.015). There was no significant difference between both cohorts with respect to gender, WHO classification, blood counts, and other blood test results [16]. AML risk groups according to the European Leukemia Net (ELN) recommendations from 2010 were equally distributed between the two cohorts (*p* = 0.923) [12]. Thirty-one (43.1%) of the AML patients with AKI suffered from AKI KDIGO stage 1, 30 (41.7%) from AKI KDIGO stage 2, and 11 (15.3%) from AKI KDIGO stage 3 (Table 2). Median days to the first AKI episode during induction chemotherapy were 18.5 days (1–120). In median, AML patients with AKI suffered from 1 AKI episode (range 1–3).

**Table 1:** Baseline characteristics

Characteristic	All	AML with AKI	AML without AKI	*p*-value
Number of patients (*n*, %)	401	72 (18)	329 (82)	
Median age (median, range)	58 (18–82)	61 (24–78)	58 (18–82)	0.014
Patients above the age of 60 (*n*, %)	174 (43.39)	37 (51.4)	137 (41.6)	0.149
Female sex (*n*, %)	188 (46.88)	32 (44.44)	156 (47.42)	0.697
Favorable ELN risk group (*n*, %)	82 (20.45)	13 (18.06)	69 (20.97)	0.923
Intermediate-I ELN risk group (*n*, %)	150 (37.41)	28 (38.89)	122 (37.08)	0.923
Intermediate-II ELN risk group (*n*, %)	91 (22.69)	16 (22.22)	75 (22.80)	0.923
Adverse ELN risk group (*n*, %)	71 (17.71)	11 (15.28)	60 (18.23)	0.923
AML with recurrent genetic abnormalities (*n*, %)	170 (42.39)	26 (36.11)	144 (43.79)	0.425
AML with myelodysplasia-related changes (*n*, %)	58 (14.46)	16 (22.22)	42 (12.77)	0.425
Therapy-related myeloid neoplasms (*n*, %)	5 (1.25)	1 (1.39)	4 (1.22)	0.425
AML not otherwise specified (*n*, %)	165 (41.15)	29 (40.28)	136 (41.34)	0.425
Albumin g/dl (median, range)*	3.8 (2.0–5.2)	3.70 (2.0–4.9)	3.8 (2.0–5.2)	0.015
White blood count/nl (median, range)*	10.71 (0.38–340.0)	8.60 (0.38–340.0)	10.90 (0.38–324.73)	0.670
Hemoglobin g/dl (median, range)*	9.10 (0.35–16.20)	8.95 (4.5–14.6)	9.10 (0.35–16.20)	0.469
Thrombocytes count/nl (median, range)*	56.00 (3–836)	51.00 (6–590)	58.00 (3–836)	0.375
Creatinine baseline mg/dl (median, range)	0.83 (0.41–1.49)	0.80 (0.48–1.45)	0.90 (0.41–1.49)	0.846
C-reactive protein mg/dl (median, range)*	2.61 (0.01–43.48)	2.69 (0.02–42.74)	2.58 (0.01–43.48)	0.243

*At time of admission to hospital. All *p*-values reported are two-sided, and statistical significance was defined as *p* ≤ 0.05

**Table 2:** Characterization of AML patients with AKI during induction chemotherapy

Characteristic	AML with AKI
Number of patients (*n*, %)	72 (18)
AKI KDIGO stage 1 (*n*, %)	31 (43.1)
AKI KDIGO stage 2 (*n*, %)	30 (41.7)
AKI KDIGO stage 3 (*n*, %)	11 (15.3)
Days to first AKI (median, range)	18.5 (1–120)
Total number of AKI (median, range)	1 (1–3)

*Median of values recorded at day of discharge. All *p*-values reported are two-sided, and statistical significance was defined as *p* ≤ 0.05

### Clinical findings in AML patients with and without AKI

There was no significant difference between the two cohorts with respect to the length of the hospital stay for induction chemotherapy (46 days vs. 49 days, *p* = 0.237) (Table 3). AML patients with AKI had a median of 7 (0–23) days with fever compared to 5 (0–31) days with fever in AML patients without AKI (*p* = 0.028). Thirty-three (45.8%) AML patients with AKI required treatment on ICU, significantly more than AML patients without AKI (*n* = 35, 10.6%, *p* < 0.001). During the hospital stay of induction chemotherapy, AML patients with AKI received 12 (0–53) RBC transfusion and 11 (2–73) platelet concentrates compared to 10 (0–35) RBC transfusion and 8 (0–33) platelet concentrates (*p* = 0.152 and *p* = 0.100 respectively). The percentage of patients receiving two cycles of induction chemotherapy was significantly lower in AML patients with AKI, (33.3% vs. 53.8%, *p* < 0.001). Thirty-four (47.2%) AML patients with AKI received allogenic SCT as consolidation therapy compared to 193 (58.7%) AML patients without AKI (*p* = 0.088). Only 39 (54.2%) AML patients with AKI achieved a CR after completion of induction chemotherapy, whereas in AML patients without AKI, 254 (77.2%) received a CR after completion of induction chemotherapy (*p* = 0.005).

**Table 3:** Clinical findings in AML patients with and without AKI

Characteristic	AML with AKI	AML without AKI	*p*-value
Number of patients (*n*, %)	72 (18)	329 (82)	
Length of hospital stay (median, range)	46 (8–127)	49 (5–125)	0.237
Days with fever (median, range)	7 (0–23)	5 (0–31)	0.028
Patients requiring treatment on intensive care unit (*n*, %)	33 (45.8)	35 (10.6)	< 0.001
Transfused RBC concentrates (median, range)	12 (0–53)	10 (0–35)	0.152
Transfused platelet concentrates (median, range)	11 (2–73)	8 (0–33)	0.100
Two cycles of induction chemotherapy (*n*, %)	24 (33.3)	177 (53.8)	< 0.001
Stem cell transplantation as consolidation therapy (*n*, %)	34 (47.2)	193 (58.7)	0.088
Complete remission after induction chemotherapy (*n*, %)	39 (54.2)	254 (77.2)	0.005
Overall mortality (*n*, %)	45 (62.5)	130 (39.5)	0.001
90-day mortality (*n*, %)	22 (30.6)	11 (3.3)	< 0.001

*Median of values recorded at day of discharge. All *p*-values reported are two-sided, and statistical significance was defined as *p* ≤ 0.05

### Laboratory findings in AML patients with and without AKI

Median hemoglobin levels and platelet counts measured throughout the hospital stay of induction chemotherapy did not differ significantly in AML patients with and without AKI (Table 4). AML patients with AKI had lower albumin levels (3.2 g/dl, range 2.1–4.0, vs. 3.4 g/dl, range 2.25–4.25, *p* < 0.001) and higher CRP levels during the hospital stay of induction chemotherapy than AML patients without AKI (5.94 mg/dl, range 0.61–34.66, vs. 3.69 mg/dl, range 0.19–87.6, *p* < 0.001). Median SCr levels during the hospital stay of induction chemotherapy were 0.83 mg/dl (0.45–3.03) in AML patients with AKI and 0.71 mg/dl (0.37–1.27) in AML patients without AKI (*p* < 0.001). SCr levels remained higher in AML patients with AKI at discharge (1.02 mg/dl, range 0.37–2.40, vs. 0.71 mg/dl, range 0.33–1.21, *p* < 0.001) and on day 90 after initiation of induction chemotherapy (0.98 mg/dl, range 0.37–1.72, vs. 0.72 mg/dl, range 0.2–3.42, *p* < 0.001).

**Table 4:** Laboratory findings in AML patients with and without AKI during the hospital stay of induction chemotherapy

Characteristic	AML with AKI	AML without AKI	*p*-value
Number of patients (*n*, %)	72 (18)	329 (82)	
Hemoglobin g/dl (median, range)	8.9 (7.40–12.00)	9 (6.40–13.15)	0.458
Platelet count/nl (median, range)	25.5 (7.0–172.0)	28 (5.5–210)	0.100
Albumin g/dl (median, range)	3.2 (2.1–4.0)	3.4 (2.3–4.3)	< 0.001
C-reactive protein mg/dl (median, range)	5.94 (0.61–34.66)	3.69 (0.19–87.60)	< 0.001
Creatinine mg/dl (median, range)	0.83 (0.45–3.03)	0.71 (0.37–1.27)	< 0.001
Creatinine at discharge mg/dl (median, range)	1.02 (0.37–2.40)	0.71 (0.33–1.21)	< 0.001
Creatinine day 90 mg/dl (median, range)	0.98 (0.37–1.72)	0.72 (0.20–3.42)	< 0.001
Highest level of procalcitonin ng/ml (median, range)	1.80 (0.09–86.00)	0.40 (0.04–159.20)	< 0.001

All *p*-values reported are two-sided, and statistical significance was defined as *p* ≤ 0.05

### Distribution of medication in AML patients with and without AKI

The distribution of anti-infective agents used in AML patients with and without AKI is illustrated in Table 5. Sixty-one (84.7%) AML patients with AKI had exposure to fluoroquinolones compared to 304 (92.4%) AML patients without AKI (*p* = 0.065). Exposure to other antibiotics (acylaminopenicillin + ß-lactamase inhibitor, carbapenems, and glycopeptides) did not differ between AML patients with and without AKI. Sixty (83.3%) AML patients with AKI received azole antifungals during the hospital stay, significantly less than 303 (92.1%) AML patients without AKI (*p* = 0.027). Liposomal amphotericin B was more often used in AML patients with AKI than in AML patients without AKI (23.6% vs. 10.9%, *p* = 0.007). Exposition to angiotensin-converting enzyme (ACE) inhibitors was significantly higher in AML patients with AKI than in AML patients without AKI (*n* = 24, 36.1%, vs. *n* = 63, 19.1%, *p* = 0.003).

**Table 5:** Distribution of medication in AML patients with and without AKI

Characteristic	AML with AKI	AML without AKI	*p*-value
Number of patients (*n*, %)	72 (18)	329 (82)	
Fluoroquinolone (*n*, %)	61 (84.7)	304 (92.4)	0.065
Acylaminopenicillin + ß-lactamase inhibitor (*n*, %)	45 (62.5)	213 (64.7)	0.786
Carbapenems (*n*, %)	57 (79.2)	259 (78.7)	1.000
Glycopeptides (*n*, %)	56 (77.8)	248 (75.4)	0.762
Azole antifungals (*n*, %)	60 (83.3)	303 (92.1)	0.027
Caspofungin (*n*, %)	27 (37.5)	110 (33.4)	0.583
Aciclovir (*n*, %)	29 (40.3)	138 (42.0)	0.895
Liposomal amphotericin B (*n*, %)	17 (23.6)	36 (11.0)	0.007
Angiotensin-converting enzyme inhibitors (*n*, %)	26 (36.1)	63 (19.1)	0.003

All *p*-values reported are two-sided, and statistical significance was defined as *p* ≤ 0.05

### Risk factors for AKI in AML patients undergoing induction chemotherapy

To further analyze risk factors for AKI in AML patients undergoing induction chemotherapy, a uni- and multivariate logistic regression model was performed. The nominal dichotome variables age above 60 years, male sex, > 5 days with fever, treatment on ICU, treatment with ACE inhibitors, and treatment with liposomal amphotericin B were included in this model. As shown in Table 6, treatment on ICU and treatment with ACE inhibitors or liposomal amphotericin B were independent risk factors for AKI in AML patients undergoing induction chemotherapy.

**Table 6:** Logistic regression analysis of risk factors for AKI

Parameter	OR	95% CI	*p*-value	OR	95 % CI	*p*-value
	Univariate analysis			Multivariate analysis		
Age > 60	1.482	0.888–2.471	0.132			
Male sex	1.127	0.675–1.882	0.647			
> 5 days with fever	1.552	0.919–2.618	0.100			
Treatment on intensive care unit	7.108	3.975–12.709	< 0.001	7.885	4.250–14.628	< 0.001
Treatment with angiotensin-converting enzyme inhibitors	2.386	1.372–4.152	0.002	2.931	1.574–5.457	0.001
Treatment with liposomal amphotericin B	2.516	1.320–4.793	0.005	2.186	1.055–4.529	0.035

Risk factors AKI were determined using a uni- and multivariate binary logistic regression model. All *p*-values reported are two-sided, and statistical significance was defined as *p* ≤ 0.05

### Outcome

**Fig. 1:**
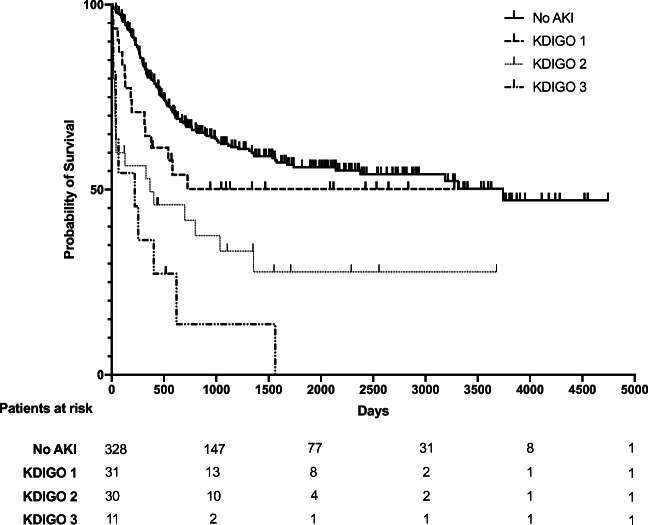
Kaplan-Meier curves for overall survival (OS). OS of AML patients without AKI (solid line) and AML patients with KDIGO stage 1 AKI (dashed line), KDIGO stage 2 AKI (dotted line), and KDIGO stage 3 AKI (dotted and dashed line)

We analyzed the survival of both patient cohorts. Overall and 90-day mortality were significantly higher in AML patients with AKI, 45 (62.5%) vs. 130 (39.5%) and 22 (30.6%) vs. 11 (3.3%) respectively (*p* = 0.001 and < 0.001 respectively) (Table 3). For AML patients without AKI during the hospital stay of induction chemotherapy, median OS was not reached. For AML patients with AKI during the hospital stay of induction chemotherapy, median OS was 402 days (95% confidence interval 87–718). We further analyzed OS with respect to the different KDIGO stages of AKI. The median OS for AML patients with AKI KDIGO stage 1 was not reached, for AML patients with KDIGO stage 2 366 days (95% confidence interval 0–1065), and for AML patients with KDIGO stage 3 220 days (95% 0–451). The Kaplan-Meier estimates with a stepwise increase of mortality with increasing AKI severity are displayed in Fig. 1.

To further analyze AKI as a prognostic parameter in AML patients undergoing intensive induction chemotherapy, a uni- and multivariate Cox regression model with forward stepwise likelihood ratio was performed. The nominal dichotome variables age above 60 years, male sex, adverse-risk AML, treatment on ICU, CR after induction chemotherapy, allogenic SCT as consolidation therapy, and AKI during induction chemotherapy were included in this model. As shown in Table 7, age above 60 years, adverse-risk AML, treatment on ICU, CR after induction chemotherapy, allogenic SCT as consolidation therapy, and AKI during induction chemotherapy were independently associated with OS. Importantly, AKI remained as an independent risk factor for OS, when censored for AKI grade 3.

**Table 7:** Univariate and multivariate analysis associated with survival in AML patients

Parameter	HR	95% CI	*p*-value	HR	95 % CI	*p*-value
	Univariate analysis			Multivariate analysis		
Age > 60	2.309	1.708–3.121	< 0.001	1.687	1.199–2.373	0.003
Male sex	1.348	1.002–1.814	0.048			
Adverse-risk AML	1.713	1.209–2.427	0.002	1.984	1.357–2.902	< 0.001
> 5 days with fever	1.216	0.902–1.640	0.200			
Treatment on intensive care unit	2.825	2.005–3.982	< 0.001	1.644	1.087–2.485	0.018
Complete remission after induction chemotherapy	0.318	0.233–0.434	< 0.001	0.415	0.297–0.581	< 0.001
Stem cell transplantation as consolidation therapy	0.394	0.292–0.533	< 0.001	0.475	0.336–0.670	< 0.001
Acute kidney injury during induction chemotherapy	2.301	1.637–3.233	< 0.001	1.761	1.184–2.619	0.005
Acute kidney injury stage 1 or 2 during induction chemotherapy	1.988	1.368–2.890	< 0.001	1.606	1.051–2.456	0.029

*CI*, confidence interval; *HR*, hazard ratio. All *p*-values reported are two-sided, and statistical significance was defined as *p* ≤ 0.05

## Discussion

During the past 30 years, advances in the management of AML patients undergoing intensified chemotherapy and bone marrow transplantation as well as improvements in supportive measures resulted in increasing survival rates [1, 2]. However, TRM remains at a level of 8–15% during induction chemotherapy [3, 4]. AKI is one of the most important organ failures associated with increased mortality and need for ICU treatment in hospitalized patients [6, 17]. To the best of our knowledge, the last investigation on the role of AKI in adult patients with AML undergoing intensive induction therapy was published in 2010, while the most recent investigation focused on pediatric AML populations [7, 8]. In this study, we evaluated risk factors for AKI in AML patients undergoing induction chemotherapy and show that AKI adversely affects the clinical course in these patients with poor CR rates after induction chemotherapy and impaired survival.

We found that AKI, defined according to the most recent KIDGO criteria, was present in 18% of AML patients undergoing induction chemotherapy. The median time to development of AKI was 18.5 days. The median time to AKI described by Lahoti et al. was also 18 days; however, the incidence of AKI was 36% significantly higher than in our study, although the RIFLE classification (used by Lahoti et al.) is more conservative than the KDIGO classification [8]. Notably, when detecting SCr increase in our study, we referred (different from Lahoti et al.) to a defined baseline SCr level from day 1 of induction chemotherapy. We thereby aimed to avoid detecting AKI by a SCr increase following on an inadequately low SCr level (e.g., due to dilution by iatrogenic fluid overload), which would be detected as AKI by Lahoti et al. but not in our study. Another explanation may be the different induction treatment protocols used in the study of Lahoti. Whereas in our study, standard induction chemotherapy was the so-called 7+3-regime; a relevant proportion of the AML population studied by Lahoti at al. received different chemotherapy regimens including the purine analogues clofarabine and fludarabine known to potentially cause AKI [18]. Finally, improvement in supportive measures in the last 10 years, e.g., by avoiding nephrotoxic drugs, is likely to contribute to the lower AKI incidence in our AML cohort.

AML patients with AKI had more days with fever than AML patients without AKI and presented with higher levels of inflammation parameters (PCT and CRP) throughout the hospital stay of induction chemotherapy. In this study, we confirmed the association of AKI and ICU treatment in AML patients as already described by others [8, 19]. Sepsis-associated inflammatory response with a potential disruption of renal perfusion as a consequence is known to be a common risk factor for AKI (https://kdigo.org/wp-content/uploads/2016/10/KDIGO-2012-AKI-Guideline-English.pdf) [6, 20].

CR is a predictor of mortality in patients with AML [7, 8]. In our investigation, we were able to show that CR rates were significantly lower in AML patients with AKI. It is obvious that the significantly lower CR rate in these AML patients cannot be simply attributed to AKI itself. The percentage of AML patients receiving two cycles of induction chemotherapy was significantly lower in all AML patients with AKI and also in AML patients with AKI younger than 60 years (regularly receiving two cycles of induction chemotherapy). We speculate that CR rates in AML patients with AKI are lower, because AML patients with AKI suffer from infectious complications and treatment on ICU. Consequently, initially intended treatment cannot be pursued or may be disrupted in AML patients with AKI. This finding is supported by the fact that patients with AKI are less likely to receive allogenic SCT as consolidation therapy and are consequently less likely to receive curative treatment. SCr values on day 90 after initiation of induction chemotherapy and at discharge from hospital remained higher in AML patients with AKI. Thus, it seems as if AKI in AML patients undergoing induction chemotherapy leads to a permanent restriction of kidney function, thereby adversely affecting the further clinical course of these patients after induction chemotherapy.

One of our key objectives was identifying risk factors associated with AKI in AML patients undergoing induction chemotherapy. Use of vancomycin and amphotericin B lipid formulation, diuretics, leukopenia, age, and hypoalbuminemia was already described as risk factors for AKI [8]. In our analysis, we confirmed the association of age with AKI in AML patients undergoing induction chemotherapy. We also found that lower albumin levels on the day of admission to hospital and especially throughout the clinical course of induction chemotherapy were associated with AKI. Decreased serum albumin levels are described to be a biomarker of poor prognosis including risk for AKI and mortality in patients treated on ICU [21–23]. Hypoalbuminemia has been described to be a predictor of mortality in different clinical setups [21, 22]. So far, albumin replacement studies in patients with sepsis and/or AKI did not improve outcomes, although the studied populations did not aim at hematological patients [24]. Prospective studies are needed to analyze whether prevention of hypoalbuminemia (e.g., by optimizing nutrition or replacing albumin) can minimize the risk for AKI in AML patients undergoing induction chemotherapy.

Potentially, nephrotoxic medications were documented for all patients and correlated with the incidence of AKI. We were able to confirm the finding of the study by Lahoti et al. regarding liposomal amphotericin B as an independent risk factor for AKI in AML patients undergoing induction chemotherapy. Furthermore, we observed a positive correlation of exposure to ACE inhibitors and AKI in AML patients undergoing induction chemotherapy. On the one hand, ACE inhibitors can cause prerenal AKI when afferent blood supply is compromised, which is present in sepsis as a result of disruption of renal perfusion [25]. Therefore, it would be of interest whether discontinuation and exchange of therapy with ACE inhibitors (e.g., by calcium channel blockers) in AML patients who are at risk of suffering from sepsis is recommendable. On the other hand, most of the AML patients taking ACE inhibitors probably have hypertensive kidney disease to some extent, thereby increasing risk for development of AKI from the beginning. The use of ACE inhibitors in prior studies was not associated with a high risk of death and was shown to be a protective factor in critically ill patients with acute renal failure [26]. Due to the retrospective nature of this study, we are not able to draw a final conclusion from this interesting finding.

In our study, we confirmed the KDIGO classifications as a prognostic model in AML patients undergoing induction chemotherapy. We were able to demonstrate a stepwise increase of mortality with increasing AKI severity. By using the KIDGO criteria, we pointed out that a relatively mild elevation in creatinine as small as 0.3 mg/dl (which might be missed in clinical practice) also adversely impacts the clinical course of these patients. We therefore advocate a sensitization not only for AKI stage 3, but for all AKI stages as we identified AKI stage 1 and 2 alone to be an independent risk factor for OS in AML patients undergoing induction chemotherapy.

In conclusion, the KIDGO classification for AKI is of high utility for mortality risk stratification in AML patients undergoing induction chemotherapy. We advocate a higher awareness of AKI, especially for mild AKI episodes which might often be overlooked. We further advise the accurate documentation of AKI episodes and the identification of patients who are prone and susceptible to AKI episodes (age, comorbidities, medication). We recommend incorporating that in the decision-making among nutrition, fluid management, the choice of antibiotic, and antifungal agents aim at decreasing the incidence of AKI. Furthermore, the early involvement of a nephrologist in patients at high risk for AKI may be reasonable for discussion of fluid management and applied medication. As to our limitations: The retrospective nature of our study limits the conclusions that can be drawn from the exhibited results. Second, our analysis did not include urine output in patients according to KDIGO criteria to classify AKI, which may have underestimated the incidence of AKI. The consideration of urine output and proteinuria is not often taken into consideration throughout AML induction therapy and should be considered as a screening and monitoring. Prospective studies would be needed to determine whether the recommended strategies will help decrease the incidence of AKI in AML patients undergoing induction chemotherapy.

## Supplementary Information

ESM 1 (DOCX 14 kb)
