# Supplementary material for: Acute kidney injury adversely affects the clinical course of acute myeloid leukemia patients undergoing induction chemotherapy
Source: Ann Hematol. 2021 Mar 11;100(5):1159–67. doi: 10.1007/s00277-021-04482-3 (PMC8043920; doi:10.1007/s00277-021-04482-3)
Supplement: Supplementary file 1 — (DOCX 14 kb) [file 277_2021_4482_MOESM1_ESM.docx]

Table S1. Staging of AKI according to KDIGO Clinical Practice Guideline 2012

| Stage | Serum creatinine | Urine output |
| --- | --- | --- |
| 1 | 1.5-1.9 times baseline OR ≥0.3 mg/dl increase | <0.5ml/kg/h for 6-12 hours |
| 2 | 2.0-2.9 times baseline | <0.5ml/kg/h for ≥12 hours |
| 3 | 3.0 times baseline OR Increase in serum creatinine to ≥4.0 mg/dl OR Initiation of renal replacement therapy OR, In patients <18 years, decrease in eGFR to <35ml/min per 1.73m^2^ | <0.3 ml/kg/h for ≥24 hours OR Anuria for ≥12 hours |
